# Editorial: Recent updates in advanced gastrointestinal endoscopy

**DOI:** 10.3389/fmed.2022.1126846

**Published:** 2023-01-05

**Authors:** Abhilash Perisetti, Benjamin Tharian, Tony C. Tham, Hemant Goyal

**Affiliations:** ^1^Department of Gastroenterology and Hepatology, Kansas City Veteran Affairs Medical Center, Kansas City, MO, United States; ^2^Digestive Health Institute, Bayfront Health St. Petersburg Medical Group, St. Petersburg, FL, United States; ^3^Division of Gastroenterology, Ulster Hospital, Dundonald, Belfast, United Kingdom; ^4^Center for Interventional Gastroenterology at UTHealth (iGUT), McGovern Medical School, University of Texas Health Science Center, Houston, TX, United States

**Keywords:** advanced endoscopy, ERCP, EUS, pancreaticobiliary cancer, advanced gastrointestinal endoscopy training

Advanced gastrointestinal endoscopy (AGE) is a subsection in the field of gastroenterology that specializes in advanced therapeutic endoscopic techniques such as complex gastrointestinal (GI) luminal, pancreatico-biliary endoscopy, and even extending beyond the lumen into third space (such as endoscopic submucosal dissection and per-oral endoscopic myotomy). With advances in optic fiber technology and endoscopy skills, gastroenterologists are positioned at the forefront of treating complex GI conditions unexplored in the past. GI cancers account for almost one-quarter of all global cancer incidence and have increased significantly in younger populations. Interventional endoscopy has a significant role in managing GI cancers, including screening, early diagnosis, and resecting lesions, thus curing them without the need for invasive surgery.

## Luminal “AGE”

In this focused issue of “*Recent updates in advanced gastrointestinal endoscopy*,” we highlight the role of advanced endoscopic techniques for luminal (first-space) esophagogastric, small intestinal, and colorectal disease states. Prevalent esophageal conditions, such as chronic gastroesophageal reflux disease (GERD), can predispose to Barrett's esophagus related neoplasia (BERN) (1). Early BERN detection with high-resolution endoscopy (chromoendoscopy, narrow-band imaging [NBI], autofluorescence, confocal laser endomicroscopy [CLE]) has revolutionized the field with a significant impact on morbidity and mortality. Multiple Enhanced endoscopic techniques for GERD (Mann, Gajendran, Perisetti, et al.) have emerged recently, such as anti-reflux mucosectomy (*via* ablation), transoral incisionless fundoplication (TIF), full-thickness plication, endostapler including gastroesophageal junction altering techniques (suturing, gastroplication, anti-reflux devices). Similarly, identification of early gastric cancer with high-magnification endoscopy (NBI, CLE) and luminal endoscopic ultrasound (EUS) can precisely stage and assist in early resection (Jiang et al.). Small bowel evaluation has been an area of limitation for endoscopists given the challenges to reach distal jejunum and ileum. With technical advances (Nehme et al.), the Sonde and Ropeway Enteroscopy have paved the way for push enteroscopy, single- and double-balloon, spiral enteroscopy, and eventually, device-assisted motorized enteroscopy. These devices are utilized in surgically altered anatomy, such as balloon-assisted (single/double) endoscopic retrograde cholangiopancreatography (ERCP) and device-assisted ERCP (Nehme et al.).

Large complex colonic polyps, which were treated surgically in the past, are now resected endoscopically using advanced polypectomy techniques such as mucosal and submucosal resection and dissection (Mann, Gajendran, Umapathy, et al.). Further, full-thickness resection devices provide an opportunity to remove early lesions in a one-step manner.

## Non-luminal “AGE”

With the advent of natural orifice transluminal endoscopic surgery (NOTES), accessing the peritoneal cavity (second space) became possible (2). However, closing the bowel wall defects remained a challenge. This led endoscopists to access the submucosal tunnel (third space) revolutionizing the field with novel techniques such as submucosal tunneling, myotomy, dissection and diverticulectomy (3). Superficial luminal GI submucosal tumors are now being treated with curative resection. In this focused issue, the efficacy and safety of myotomy in sigmoidization of esophagus in achalasia is noted with good clinical and technical success with low rate of adverse events (Xu et al.). The role of EUS has been extended from diagnostic to therapeutics such as peripancreatic fluid drainage, EUS-guided biliary drainage, EUS-guided pancreatic duct drainage, transmural access EUS-guided gastrojejunostomy, EUS-guided celiac plexus neurolysis or block, EUS-guided liver biopsy (4). EUS has also helped us access vascular structures such as (5) gastric varices and portal vessels for variceal coiling and portal pressure monitoring. Use of injection therapy has allowed us to perform EUS-guided anti-tumor therapy such as ethanol for pancreatic neuroendocrine tumors and local radiofrequency ablation for unresectable pancreatic tumors (Yousaf et al.). Finally, development of over-the-scope-clips (OTSC), lumen apposing and non-lumen apposing stents are helping endoscopists to diagnose and manage common complications such as bleeding, perforation and fistulous tracts.

## The future of “AGE”

With emergence of aforementioned techniques, the field of AGE seems to be optimistic and promising for further novel approaches. Artificial intelligence (Xiao et al.) has unfolded predictive capacity of detecting precancerous and cancerous lesions (Fu et al.) with machine learning and convolutional neural network (6–8). Automated polyp characterization (size, optical pathology), cecum detection, bowel preparation scoring and esophagogastric neoplasia detection (Jiang et al.) is now possible with the use of AI. AGE is currently seeing an unprecedented progress with AI to predict, detect and manage neoplastic lesions with a precision which was never imagined. AGE could potentially use robotic techniques (9) such as robotic flexible endoscopy, forceps manipulation, transluminal access, neoguide endoscopic system and endoscopic capsules. However, with all of these techniques, there is considerable learning curve for which extensive training and research is needed to assess the intricacies, determine the pathway for appropriate credentialing and reporting of adverse events. This could be performed using quality (Song et al.) metrics as a cornerstone for any procedure with its outcomes. Further, given the complexity of the endoscopic work, a collaborative effort with interventional radiology and surgical teams can bring out the best outcomes.

## Author contributions

AP: topic overview, outline, idea generation, writing, and editing. HG: critical editing, idea generation, and finalizing the article. TT and BT: finalizing the article. All authors contributed to the article and approved the submitted version.
